# Deep Learning in Automatic Sleep Staging With a Single Channel Electroencephalography

**DOI:** 10.3389/fphys.2021.628502

**Published:** 2021-03-03

**Authors:** Mingyu Fu, Yitian Wang, Zixin Chen, Jin Li, Fengguo Xu, Xinyu Liu, Fengzhen Hou

**Affiliations:** ^1^School of Science, China Pharmaceutical University, Nanjing, China; ^2^College of Engineering, University of California, Berkeley, Berkeley, CA, United States; ^3^College of Physics and Information Technology, Shaanxi Normal University, Xi’an, China; ^4^Key Laboratory of Drug Quality Control and Pharmacovigilance, China Pharmaceutical University, Nanjing, China

**Keywords:** deep learning, single channel electroencephalography, automatic sleep staging, bidirectional long short-term memory, attention mechanism

## Abstract

This study centers on automatic sleep staging with a single channel electroencephalography (EEG), with some significant findings for sleep staging. In this study, we proposed a deep learning-based network by integrating attention mechanism and bidirectional long short-term memory neural network (AT-BiLSTM) to classify wakefulness, rapid eye movement (REM) sleep and non-REM (NREM) sleep stages N1, N2 and N3. The AT-BiLSTM network outperformed five other networks and achieved an accuracy of 83.78%, a Cohen’s kappa coefficient of 0.766 and a macro F1-score of 82.14% on the PhysioNet Sleep-EDF Expanded dataset, and an accuracy of 81.72%, a Cohen’s kappa coefficient of 0.751 and a macro F1-score of 80.74% on the DREAMS Subjects dataset. The proposed AT-BiLSTM network even achieved a higher accuracy than the existing methods based on traditional feature extraction. Moreover, better performance was obtained by the AT-BiLSTM network with the frontal EEG derivations than with EEG channels located at the central, occipital or parietal lobe. As EEG signal can be easily acquired using dry electrodes on the forehead, our findings might provide a promising solution for automatic sleep scoring without feature extraction and may prove very useful for the screening of sleep disorders.

## Introduction

Sleep is important for the optimal functioning of the brain and the body (Czeisler, 2015). However, a large number of people suffer from sleep related disorders, such as sleep apnea, insomnia and narcolepsy (Ohayon, 2002). Effective and feasible sleep assessment is essential for recognizing sleep problems and making timely interventions.

Sleep assessment is generally based on the manual staging of overnight polysomnography (PSG) signals, including electroencephalogram (EEG), electrooculogram (EOG), electromyogram (EMG), electrocardiogram (ECG), blood oxygen saturation and respiration (Weaver et al., 2005), by trained and certified technicians. According to the American Academy of Sleep Medicine (AASM) manual (Iber et al., 2007), sleep can be staged as wakefulness (WAKE), rapid eye movement (REM) sleep and non-REM (NREM) sleep, which is further divided into three stages, N1, N2 and N3. Usually, it takes about 2–4 h for a technician to mark an overnight (lasting about 8 h) PSG. The time-consuming nature of manual sleep staging hampers its application on very large datasets and limits related research in this field (Hassan and Bhuiyan, 2016a). Moreover, the inter-scorer agreement is less than 90% and its improvement remains a challenge (Younes, 2017). The multiple channels of PSG also present drawbacks preventing wider usage for the general population, due to complicated preparation and disturbance to participants’ normal sleep. Therefore, the past decades have witnessed the growth of automatic sleep staging based on single-channel EEG (Liang et al., 2012; Ronzhina et al., 2012; Aboalayon et al., 2014; Radha et al., 2014; Zhu et al., 2014; Wang et al., 2015; Hassan and Bhuiyan, 2016a, 2017; Boostani et al., 2017; Phan et al., 2017; Silveira et al., 2017; Tian et al., 2017; Lngkvist and Loutfi, 2018; Seifpour et al., 2018; Sors et al., 2018; Tripathy and Acharya, 2018). These methods may eventually lead to a sufficiently accurate, robust, cost-effective and fast means of sleep scoring (Wang et al., 2015).

In the field of machine learning, deep networks are drawing more and more attention because they can learn from data directly without manual feature extraction (Lecun et al., 2015; Tsinalis et al., 2015; Dong et al., 2016; Supratak et al., 2017; Zhang and Wu, 2017; Bresch et al., 2018; Malafeev et al., 2018; Stephansen et al., 2018). There are many useful and well-established deep networks for the data mining of time series, such as the convolutional neural network (CNN) (Lecun and Bengio, 1997) and recurrent neural network (RNN) (Elman, 1990). Although CNN has mainly been applied in automated recognition of images, its application in the analysis of time series has also been notable (Chambon et al., 2018; Cui et al., 2018; Zhang and Wu, 2018; Yildirim et al., 2019). That said, it is generally demonstrated that RNN has better performance than CNN for the analysis of time series (Fiorillo et al., 2019). One of the most widely used RNN is the Long Short-Term Memory (LSTM) neural network, which is capable of capturing the long-term dependent information underlying the temporal structure of the time series (Hochreiter and Schmidhuber, 1997). Furthermore, bidirectional LSTM (BiLSTM), composed of two unidirectional LSTMs, can read data from both ends of the time series and is able to make full use of information embedded in both directions of the time series (Schuster and Paliwal, 1997). Moreover, the concept of attention is arguably one of the most powerful in the deep learning field nowadays. It is based on a common sense intuition that we “attend to” a certain part when processing a large amount of information. This simple yet powerful concept has led to many breakthroughs, not only in natural language processing tasks, such as speech recognition (Jo et al., 2010) and machine translation (Ferri et al., 2012; Karpathy and Fei-Fei, 2014; Hassan and Bhuiyan, 2017), but also in time series analysis. Recently, Zhang et al. (2019) proposed an attention-based LSTM model for financial time series prediction and a comparative analysis conducted by Hollis et al. (2018) further demonstrates that an LSTM with attention indeed outperforms a standalone LSTM for forecasting financial time series.

The application of deep neural networks for automatic sleep staging is soaring (Table 1). The PhysioNet Sleep-EDF Expanded (PSEE) dataset (Goldberger et al., 2000; Kemp et al., 2000) was the most widely employed dataset in related studies. As shown in Table 1, Tsinalis et al. (2016) and Phan et al. (2019) reported an accuracy of 74.0% and 81.9% respectively, for 5-class sleep staging of the PSEE dataset with a CNN algorithm, while Supratak found that the combination of CNN and BiLSTM increased the accuracy to 82.4% (Supratak et al., 2017). There are also some datasets aside from PSEE that are routinely employed in studies of automatic sleep staging with a single-channel EEG and deep learning algorithms. Hsu et al. (2013) built an RNN model on the PhysioNet Sleep-EDF (PSE) dataset and achieved an accuracy of 87.2%. On the Montreal Archive of Sleep Studies (MASS) dataset, Phan et al. (2019) built a CNN model and achieved an accuracy of 83.6% while Supratak et al. (2017) built a CNN-LSTM model and obtained an accuracy of 86.2%. A CNN was also applied on the Sleep Heart Health Study (SHHS) dataset, yielding an accuracy of 87% (Sors et al., 2018). However, few works investigated whether the performance of sleep staging can be further improved by the combination of BiLSTM and the attention mechanism. Aside from that, there is a lack of comparison between the performance of deep learning based and conventional feature extraction based models.

**TABLE 1:** An overview of the application of deep networks on sleep staging.

Authors	Dataset	Channel	Model	Accuracy
Tsinalis et al.	PSEE	Fpz-Cz	CNN	74.0%
Phan et al.	PSEE	Fpz-Cz	CNN	81.9%
Supratak et al.	PSEE	Fpz-Cz	CNN-BiLSTM	82.4%
Hsu et al.	PSE	Fpz-Cz	RNN	87.2%
Phan et al.	MASS	C4-A1	CNN	83.6%
Supratak et al.	MASS	F4-EOG (Left)	CNN-BiLSTM	86.2%
Sors et al.	SHHS	C4-A1	CNN	87.0%

Although deep learning algorithms have shown themselves promising in automatic sleep staging with a single-channel EEG, few studies investigated whether the performance of such algorithms is sensitive to the choice of EEG channel. Therefore, in this study, the PSEE dataset and the DREAMS Subjects (DRM-SUB) dataset (Devuyst, 2005) were used. Both datasets have more than one channel of EEG and the DRM-SUB dataset was involved in many automatic sleep staging studies with conventional feature extraction (Hassan and Bhuiyan, 2016a, 2017; Ghimatgar et al., 2019; Shen et al., 2019). A neural network named AT-BiLSTM was proposed, which uses the neural attention mechanism of the BiLSTM to classify sleep stages. For comparison, five other networks, CNN, LSTM, BiLSTM, the combination of CNN and LSTM (CNN-LSTM), and the combination of CNN and BiLSTM (CNN-BiLSTM) were also trained and tested. Our aims are threefold: first, to investigate whether AT-BiLSTM can achieve the highest performance among these networks; second, to confirm whether RNN algorithms (i.e., LSTM and BiLSTM) outperform CNN in sleep staging with single channel EEG; third, to explore whether the method of making hybrid networks further improves the performance of sleep staging.

## Materials and Methods

### Datasets

The data analyzed in this study were obtained from two open-access datasets: the DRM-SUB dataset and the PSEE dataset. The DRM-SUB consists of 20 whole-night PSG recordings (lasting 7–9 h) obtained from 20 subjects (four males and 16 females, 20–65 years old). Three EEG channels located in different lobes (Cz-A1, Fp1-A1 and O1-A1) were included in DRM-SUB, with a sampling rate of 200 Hz. To investigate the impact of the choice of EEG derivations on the performance of automatic sleep staging, EEG signals from all three channels were used separately for the following analysis.

Twenty healthy subjects (10 males and 10 females, 25–34 years old) from the PSEE dataset were also included. There are two EEG channels (Fpz-Cz and Pz-Oz) available in the PSEE dataset, with a sampling rate of 100 Hz. For each subject, two PSGs of about 20 h each were recorded during two subsequent day-night periods at the subjects’ homes. In order to remain consistent with previous studies (Supratak et al., 2017), for each subject and each PSG, only the data from 30 min before sleep-onset (i.e., the first sleep epoch after light-off in the evening) and 30 min after the last sleep epoch in the morning were included. Both channels were investigated separately.

For both datasets, labels of sleep staging for each 30-s EEG epoch were provided by the data distributors according to AASM rules. Five staging classes, i.e., WAKE, N1, N2, N3, and REM were used in this study. The distribution of 30-s EEG epochs of both datasets is illustrated in Table 2.

**TABLE 2:** Data distribution of sleep stages in both datasets.

Dataset	Total epochs	WAKE (%)	N1 (%)	N2 (%)	N3 (%)	REM (%)
PSEE	41663	19.2	6.6	42.2	13.4	18.5
DRM-SUB	20265	17.6	7.3	40.7	19.4	14.9

### Construction of the AT-BiLSTM Network

**FIGURE 1:**
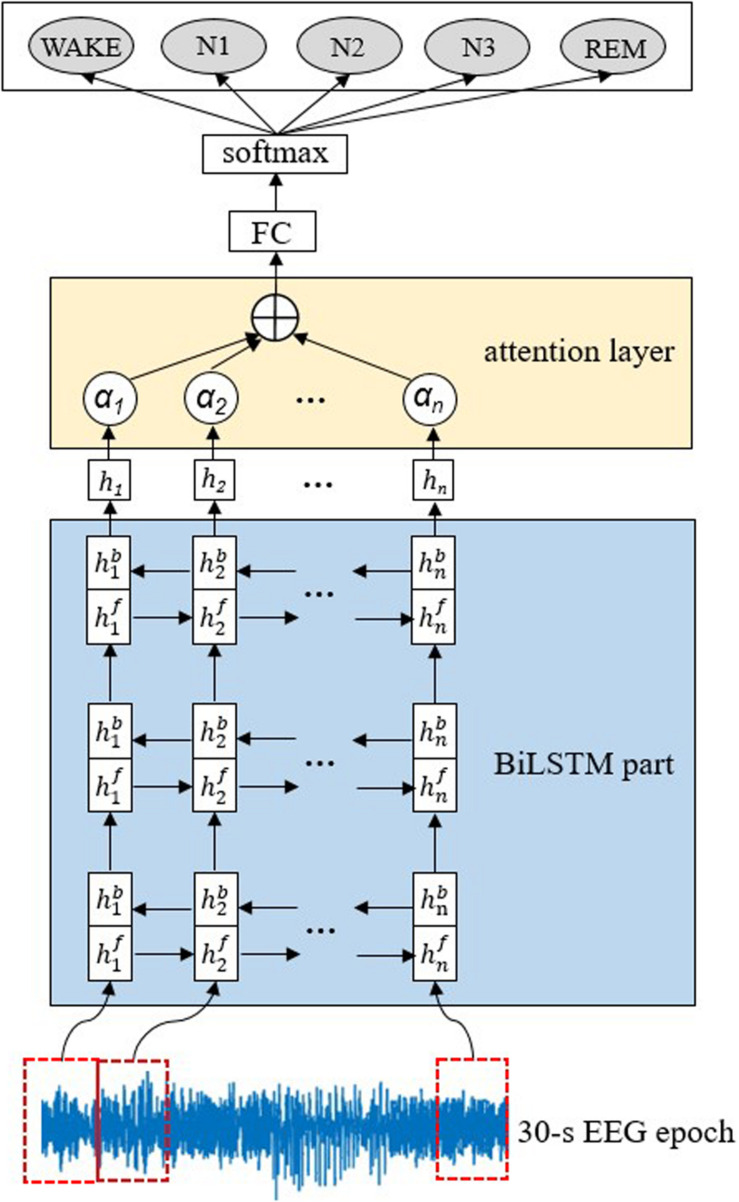
Illustration of the proposed AT-BiLSTM network architecture for automated sleep staging. The network consists of a BiLSTM part, an attention layer, a full-connected (FC) layer and a softmax layer. The input of the network is a raw 30-s EEG time series and the output is the probability of each sleep stage. The dashed rectangle on the EEG time series represents a vector of EEG signals at a time step.

The proposed AT-BiLSTM network architecture for automatic sleep staging is illustrated in Figure 1. It is composed of two main components, three stacked BiLSTM layers for feature exacting and one attention layer to weight the most relevant parts of the input sequence. According to a preset parameter, called the input dimension *m*, each raw 30-s EEG epoch is divided into multiple vectors, which are fed into the BiLSTM part sequentially to construct a feature matrix. Then to emphasize the different importance of different vectors, an attention layer is applied in the intra-epoch feature learning and summarizes the outputs of the BiLSTM part with different weights. Finally, the probability of each sleep stage can be derived from a fully connected (FC) layer and a softmax layer.

Given a 30-s EEG epoch *X*[*x*_1_,*x*_2_,,*x_N_*] with *N* data points, a moving window with input dimension of *m* is applied to **X** without overlap, leading to the matrix form of **X**, as shown in Equation 1, where *n* equals to *N/m* and **X_t_** represents the vector in time step *t*.

(1) $$\left[\begin{array}{c} {\text{X}}_{1}\\ {\text{X}}_{2}\\ \vdots \\ {\text{X}}_{t}\\ \vdots \\ {\text{X}}_{n} \end{array}\right]=\left[\begin{array}{cccc} x_{1} & x_{2} & \cdots & x_{m}\\ x_{m+1} & x_{m+2} & \cdots & x_{2m}\\ \vdots & \vdots & \cdots & \vdots \\ x_{\left(t-1\right)m+1} & x_{\left(t-1\right)m+2} & \cdots & x_{t\times m}\\ \vdots & \vdots & \cdots & \vdots \\ x_{\left(n-1\right)m+1} & x_{\left(n-1\right)m+2} & \cdots & x_{n\times m} \end{array}\right]t\in \left[1,n\right]$$

All the vectors are fed into the first BiLSTM layer, forward and backward respectively. For time step *t*, the output of the forward or backward network, denoted as ${\text{h}}_{t}^{f}$ or ${\text{h}}_{t}^{b}$, can be obtained, respectively, according to Equation 2 or 3.

(2) $${\text{h}}_{t}^{f}=\sigma \left({\text{W}}_{\mathrm{fx}}{\text{x}}_{t}+{\text{W}}_{\mathrm{ff}}{\text{h}}_{t-1}^{f}+b_{f}\right)$$

(3) $${\text{h}}_{t}^{b}=\sigma \left({\text{W}}_{\mathrm{bx}}{\text{x}}_{t}+{\text{W}}_{\mathrm{bb}}{\text{h}}_{t-1}^{b}+b_{b}\right)$$

where σ is the logistic sigmoid function, **W** is the weight matrix (e.g., subscription “*fx*” in **W** represents the forward network of **x_t_**) and *b* is the bias vector of the network (*bf* and *bb* represents the bias vector of forward and backward network, respectively).

The weighted sum of ${\text{h}}_{t}^{f}$ and ${\text{h}}_{t}^{b}$, denoted as **h_t_**, is computed as the output of the first BiLSTM layer following Equation 4.

(4) $${\text{h}}_{t}={\text{W}}_{\mathrm{hf}}{\text{h}}_{t}^{f}+{\text{W}}_{\mathrm{hb}}{\text{h}}_{t}^{b}+b_{h}$$

The output of the previous BiLSTM layer is fed into the next layer in the same way. The third layer gives the final output of the BiLSTM part, which is weighted by the attention layer before feeding into the FC layer. Considering that EEG signal in different time steps should contribute differently to the classification task, it is rational to give strong weights to the more discriminative parts and vice versa. Formally, the attention weight **a_t_** at the time step *t* is computed according to Formula (5) – (6).

(5) $$u_{t}=\tanh\left(w_{w}h_{t}+b_{w}\right)$$

(6) $$a_{t}=\frac{\text{exp}\left(u_{t}^{T}u_{w}\right)}{\sum_{t}\text{exp}\left(u_{t}^{T}u_{w}\right)}$$

In Formula (5)–(6), **u_t_** represents the state of the hidden layer obtained from a simple neural network, **u_w_** represents a weight vector randomly initialized, **a_t_** represents the similarity between **u_t_** and **u_w_** obtained by softmax function.

(7) $${\text{s}}_{t}=\sum_{t}{\text{a}}_{t}{\text{h}}_{t}$$

By weighting and summing the output of the BiLSTM part, the attention vector, denoted as **s_t_**, can be obtained and fed into FC layer, preceding to the softmax layer which finally yields the probability of each sleep stage.

### Construction of Baseline Networks

Apart from the proposed AT-BiLSTM network, we also constructed five baseline networks, including three single networks, i.e., CNN, LSTM and BiLSTM, and two hybrid networks, i.e., CNN-LSTM and CNN-BiLSTM.

### Single Networks

**FIGURE 2:**
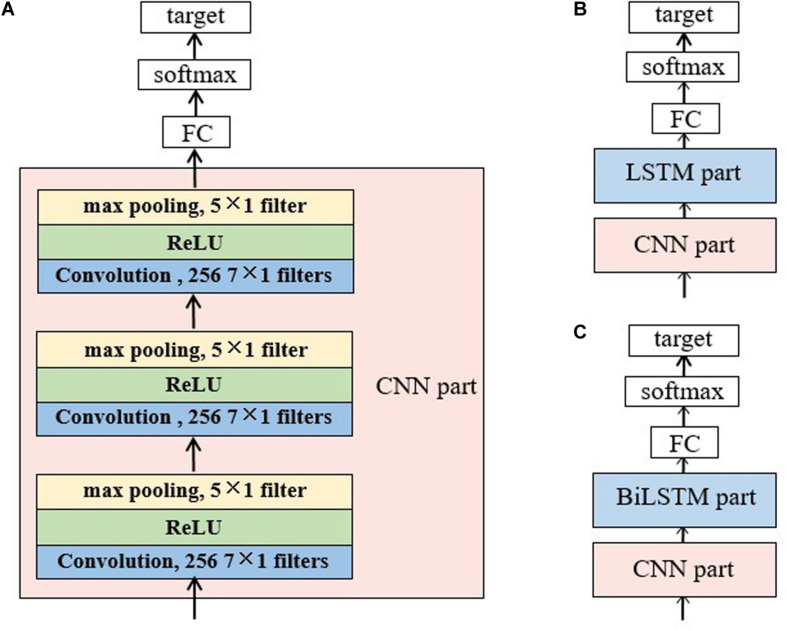
Structure of the baseline networks for sleep staging: **(A)** the CNN network, **(B)** the CNN-LSTM network and **(C)** CNN-BiLSTM network. The CNN network consists of a CNN part, a full-connected layer and a softmax layer. In the CNN part, there are three convolution layers and three max pooling layers. Each convolution layer has 256 filters with a size of 7 × 1 each and each pooling layer has one filter of size 5 × 1. A rectified linear unit (ReLU) follows the convolution layer and precedes the pooling layer. The CNN part in panels **(B,C)** has the same topology with panel (A). For the LSTM/BiLSTM part, there are three stacked LSTM/BiLSTM layers with each layer consists of 256 memory cells. The target for all the networks was the probability of each sleep stage.

Figure 2A illustrated the CNN topology used in this study, which is fed with a matrix reconstructed from a raw 30-s EEG epoch according to Equation 1. It consists of three convolution blocks and three max pooling layers. Each convolutional block contains a one-dimensional convolutional layer and a rectified linear unit (ReLU) activation layer. The input matrix is padded with zeros to ensure that the number of rows in the matrix is constant during the convolutional process. The output of CNN is fed into a FC layer, then activated by softmax function to obtain the sleep stage probability.

Two scenarios were considered in single RNN network. In the first scenario, three layers of LSTMs were stacked, also followed by a FC layer and a softmax layer. The second scenario employed stacked BiLSTM layers instead of the LSTM layers.

### Hybrid Networks With CNN and RNN

As shown in Figure 2B,C, a CNN part followed by an RNN part was adopted in the hybrid networks, in order to make use of RNN for further processing the features extracted by CNN. The structures of the CNN part and RNN part are the same with the single networks aforementioned.

### Datasets Splitting Strategy

**FIGURE 3:**
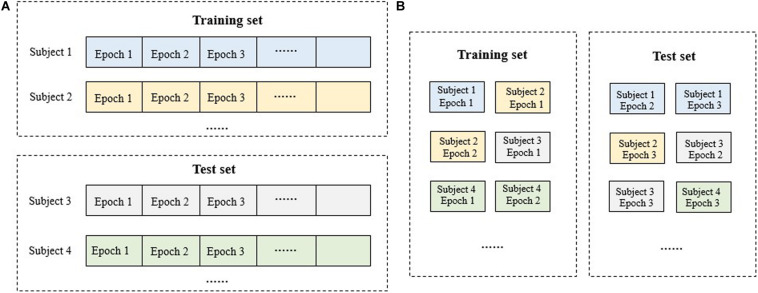
Schematic diagram for the dataset splitting of training and test set: **(A)** subject-wise method; **(B)** epoch-wise method. For the subject-wise method, all the 30-s EEG epochs from a subject will be included in the training set or the test set as a whole while for the epoch-wise method, the epochs of a subject may appear in both the training and test set.

Machine learning algorithms require independent training and test sets for model training and performance evaluation. Also, k-fold cross validation is preferred in application. Generally, there are two types of training data partitioning for clinic data: subject-wise and epoch-wise (Figure 3). For the subject-wise method, all the subjects were split into k folds equally and onefold is taken as the test set in turn while the remains as the training set. For the epoch-wise method, all the 30-s EEG epochs from all the subjects were merged and then split into k equal folds for each stage randomly. That is, for each sleep stage, all the 30-s EEG epochs from all the subjects were collected and divided into k folds. Consequently, the epochs of a subject may appear in both the training and test set, violating the independence between the training and test set and contributing to a virtual high performance. Thus, in the present study, the subject-wise method with fivefold cross validation was adopted. The model was trained using the training set and evaluated using the test set. Finally, all evaluation results were combined.

### Experimental Setting and Network Optimization

Using the first fold as the test set, the network parameters, such as the input dimension, the number of hidden units in each LSTM/BiLSTM/convolutional layer, and the filter/stride size of each convolutional layer and pooling layer, were determined by a grid-search to minimize the errors of networks with Python 3.6 and TensorFlow v1.15.0 (Abadi et al., 2016). The standard cross-entropy loss was used as the loss function in model training due to its good performance in measuring the errors of networks with discrete targets (Boer et al., 2005). Each network was trained for 30 epochs with a mini batch size of 64 sequences. As a result, the input dimension *m* was set as 5, the number of hidden units as 256, and the stride size for both convolution layers and max pooling layers as 1 × 1. The filter size of each convolutional layer and max pooling layer in CNN were set to 1 × 7 and 1 × 5 respectively.

For backpropagation, the adaptive moment estimation (ADAM) algorithm was adopted because it solves the optimization problem in non-stationary conditions and works faster than the standard gradient descent algorithm and the root mean square propagation (Kingma and Ba, 2017). The main hyper-parameters used for ADAM algorithm were set as: learning rate (α = 0.001), gradient decay factor (β1 = 0.9), squared gradient decay factor (β2 = 0.999), and epsilon (ε = 10–8) for numerical stability. Moreover, a dropout layer before the last FC layer was used to avoid over-fitting and its dropout rate was set to 0.2, leading to 20% of the weights dropped during the training phase.

### Performance Metrics

Overall metrics, including accuracy, macro F1-score (MF1) and Cohen’s kappa (κ) were used to evaluate the performance of each model. Performance on individual sleep stages was also assessed *via* class-wise precision and sensitivity.

Cohen’s kappa coefficient is a statistical measure of inter-rater agreement for categorical items (Cohen, 1960). When two persons (algorithms or raters) try to evaluate the same data, Cohen’s Kappa coefficient, κ, is used as a measure of agreement between their decisions. In this study, it measures the amount of agreement between the output of the proposed algorithm and the provided labels of sleep stages.

Another metric used for performance evaluation here is the area under the receiver operating characteristics (ROC) curve, called AUC. The ROC curve is a graphical tool and demonstrates the classification performance by plotting the true positive rate (TPR) against the false positive rate (FPR) at different classification thresholds (Zweig and Campbell, 1993). Furthermore, it provides a convenient way for selecting the threshold that provides the maximum classification TPR while not exceeding a maximum allowable FPR level (Kim et al., 2019). For an *n*- class classification task, *n* ROC curves can be obtained by splitting the task into *n* binary classification tasks. For each binary classification task, its AUC value can be used as a class-wise measure of performance and the macro-average AUC of these tasks can be regarded as an overall metric for the performance evaluation.

## Results

Table 3 shows the overall performance of different networks on the PSEE dataset. The proposed AT-BiLSTM network outperforms other networks with overall accuracy, κ, MF1 and MAUC of 83.78%, 0.766, 82.14% and 97.45% on channel Fpz-Cz, respectively and an overall accuracy, κ, MF1 and MAUC of 80.79%, 0.731, 79.27% and 96.33% on channel Pz-Oz, respectively. The AT -BiLSTM network performs better than the other networks overall. For the single networks, the RNN-based networks outperform the CNN network while the results of BiLSTM and LSTM are comparable. The hybrid networks further improve the overall performance compared to the single models. Moreover, AT-BiLSTM achieves better precision and sensitivity on N3 and REM than the hybrid networks with CNN and RNN, although they have a comparable performance on stages Wake, N1 and N2. Furthermore, better performance is found in Fpz-Cz than Pz-Oz channel, regardless of the network topology used, indicating EEG derived from the frontal lobe is more valuable than those from the parietal lobe in sleep staging.

**TABLE 3:** The overall performance of different networks on the PSEE dataset (value in bold represents for the best among all the networks).

Networks	Fpz-Cz				Pz-Oz			
	Acc.	κ	MF1	AUC	Acc.	κ	MF1	AUC
AT-BiLSTM	**83.78**	**0.766**	**82.14**	**96.08**	**80.79**	**0.731**	**79.27**	**93.63**
CNN	78.84	0.706	76.10	92.89	76.45	0.669	74.56	89.91
LSTM	81.59	0.747	79.25	95.36	79.02	0.706	75.92	92.14
BiLSTM	81.48	0.740	80.13	93.78	78.95	0.707	77.44	91.81
CNN-LSTM	82.58	0.759	80.40	93.96	79.51	0.718	76.44	92.36
CNN-BiLSTM	82.58	0.759	81.15	94.67	79.37	0.710	77.92	92.70

Table 4 shows the performance of different networks on the DRM-SUB dataset. The AT -BiLSTM network still outperforms other networks, suggesting its good generalization in sleep staging. Consistent with the results in PSEE dataset, the frontal EEG channel (Fp1-A1 here) achieves the best performance. The results are in line with a recent work, which found that EEG signals from an Fp1-A1 channel yielded higher accuracy values in automatic sleep staging than those of a Cz-A1 or O1-A1 channel (Ghimatgar et al., 2019).

**TABLE 4:** The overall performance of different networks on the DRM-SUB dataset (value in bold represents for the best among all the networks).

Networks	Fp1-A1				Cz-A1				O1-A1			
	Acc.	κ	MF1	AUC	Acc.	κ	MF1	AUC	Acc.	κ	MF1	AUC
AT-BiLSTM	**81.72**	**0.751**	**80.74**	**94.99**	**81.62**	**0.749**	**80.76**	**95.25**	**77.09**	**0.685**	**75.98**	**94.91**
CNN	77.84	0.732	67.17	89.90	75.82	0.664	73.98	91.00	72.45	0.617	70.84	90.59
LSTM	80.19	0.738	70.96	94.29	80.53	0.733	80.23	94.65	74.13	0.641	72.32	92.97
BiLSTM	80.31	0.739	70.41	93.84	80.41	0.733	79.58	94.66	74.22	0.644	72.98	92.85
CNN-LSTM	80.55	0.738	71.96	94.43	80.87	0.736	79.24	94.50	75.78	0.665	74.52	93.73
CNN-BiLSTM	80.61	0.737	71.62	93.87	80.83	0.738	80.71	94.68	75.94	0.666	74.42	93.79

**FIGURE 4:**
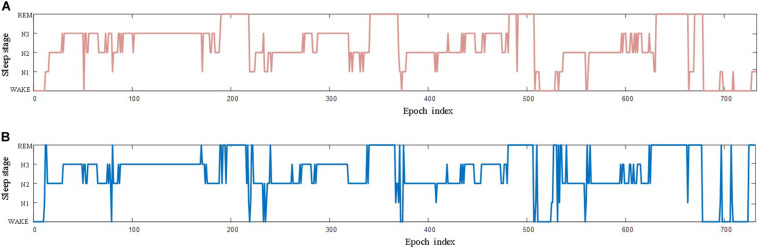
The hypnograms labeled **(A)** manually by a clinical technician of sleep and **(B)** by the trained AT-BiLSTM model. The corresponding EEG recoding was obtained from PSEE dataset (SC4001E0).

Figure 4 illustrates the hypnograms labeled manually by a clinical technician of sleep and by the trained AT-BiLSTM model. The corresponding EEG recoding was obtained from the first person in PSEE dataset (SC4001E0), who spent 7 h during sleep. Noting that the subject is located in the test set for the trained model. The accuracy of the automatic sleep staging for this subject is 87.30%, showing considerable reliability of the proposed AT-BiLSTM network. Most of the wrong classifications were made during the transitions from one stage to another.

Table 5 shows the class-wise performances obtained on the PSEE dataset. For most stages, better performance is achieved by the AT-BiLSTM model than the baseline networks and Fpz-Cz channel outperforms the Pz-Oz one. Although the classification accuracy of stage N1 is significantly lower than that of the other stages, which might due to the small percentage of N1 during sleep, it is higher than those reported in previous studies (Hsu et al., 2013; Supratak et al., 2017). Similar findings can be found on the DRM-SUB (Table 6).

**TABLE 5:** The class-wise performance obtained on the PSEE dataset (value in bold represents for the best among all the networks).

EEG signal	Networks	Precision					Sensitivity					Class-wise AUC				
		W	N1	N2	N3	REM	W	N1	N2	N3	REM	W	N1	N2	N3	REM
**Fpz-Cz**	AT-BiLSTM	**86.38**	**45.06**	87.82	**88.88**	**76.84**	89.42	**25.76**	**89.18**	**89.62**	**82.18**	97.58	**88.85**	**97.31**	**99.34**	**97.33**
	CNN	84.68	34.32	84.78	82.66	67.08	83.88	19.34	84.98	82.63	77.22	95.98	82.00	94.18	97.99	94.32
	LSTM	84.72	43.28	85.98	88.68	71.92	87.74	20.68	88.16	85.12	80.14	**98.72**	84.85	97.25	99.08	96.94
	BiLSTM	81.34	41.23	86.95	86.71	69.90	88.32	20.84	86.88	89.42	81.43	96.70	81.18	96.54	99.04	95.45
	CNN-LSTM	85.72	45.16	**88.46**	87.22	71.04	**90.14**	12.46	86.54	88.46	79.22	96.52	81.29	96.77	99.17	96.09
	CNN-BiLSTM	85.16	42.51	87.56	87.42	75.72	88.72	25.34	87.42	88.64	79.76	97.19	83.26	96.96	99.22	96.74
**Pz-Oz**	AT-BiLSTM	**82.58**	40.24	**84.64**	**84.84**	**71.76**	**82.58**	40.24	**84.64**	**84.84**	**71.76**	**96.23**	**82.54**	**96.08**	**98.76**	**94.52**
	CNN	78.48	24.18	81.16	79.42	63.28	78.48	24.18	81.16	79.42	63.28	94.59	75.96	92.70	95.17	91.14
	LSTM	79.84	41.82	82.94	82.36	64.82	79.84	41.82	82.94	82.36	64.81	95.34	78.34	94.80	98.35	93.88
	BiLSTM	80.64	42.94	83.78	82.26	66.70	80.64	42.94	84.28	82.26	66.74	95.19	76.55	95.16	98.46	93.69
	CNN-LSTM	80.95	42.65	83.95	82.55	69.75	80.95	42.65	83.95	82.55	69.75	95.31	78.72	95.26	98.48	94.04
	CNN-BiLSTM	79.52	**44.62**	84.26	83.04	70.37	79.55	**44.62**	84.26	83.04	70.38	95.79	80.53	95.16	98.39	93.63

**TABLE 6:** The class-wise performance obtained on the DRM-SUB dataset (value in bold represents for the best among all the networks).

EEG signal	Networks	Precision					Sensitivity					Class-wise AUC				
		W	N1	N2	N3	REM	W	N1	N2	N3	REM	W	N1	N2	N3	REM
**Fp1-A1**	AT-BiLSTM	**88.48**	45.92	**84.98**	**89.08**	**68.26**	89.34	**25.88**	**85.12**	85.18	**83.06**	**99.50**	**88.80**	**93.05**	**96.69**	**96.93**
	CNN	83.54	40.82	79.76	87.14	63.02	84.22	11.54	85.92	78.52	78.96	97.85	82.77	87.05	86.85	94.96
	LSTM	83.63	44.16	82.58	88.26	68.14	89.92	19.18	84.82	84.74	75.28	99.35	87.06	92.33	96.40	96.33
	BiLSTM	85.24	43.82	83.28	86.82	66.28	86.78	17.36	85.31	86.83	77.74	98.90	85.83	92.01	96.39	96.07
	CNN-LSTM	87.12	42.26	84.52	86.26	66.44	87.04	23.34	84.52	**87.66**	79.91	99.26	88.22	91.94	96.61	96.13
	CNN-BiLSTM	86.62	**46.92**	83.88	86.74	66.38	**89.98**	20.74	84.54	85.88	79.86	99.21	86.30	92.04	96.28	95.54
**Cz-A1**	AT-BiLSTM	**88.02**	42.02	**84.98**	87.56	69.22	90.96	22.22	86.32	**86.28**	**80.92**	**99.40**	**89.24**	**93.62**	**97.10**	**96.87**
	CNN	83.68	23.44	75.18	86.14	65.12	86.55	8.92	85.36	73.28	72.68	98.18	82.81	88.39	90.85	94.77
	LSTM	87.24	43.88	83.44	87.76	69.26	89.54	22.34	86.12	86.26	77.04	99.14	88.59	92.39	97.06	96.09
	BiLSTM	86.46	39.68	83.82	88.58	67.12	89.24	20.62	85.36	83.38	78.42	99.27	88.56	92.57	96.77	96.14
	CNN-LSTM	84.34	**48.72**	81.44	**89.58**	70.64	**91.34**	14.16	87.66	82.46	78.06	98.99	88.43	92.42	96.49	96.15
	CNN-BiLSTM	86.22	42.96	82.32	87.98	**70.88**	90.36	**23.68**	**86.78**	84.26	76.58	99.30	88.68	92.48	96.66	96.29
**O1-A1**	AT-BiLSTM	88.86	**46.58**	**78.76**	83.20	**62.56**	**91.36**	**18.53**	81.94	78.67	**72.74**	**99.55**	**89.76**	**92.40**	**96.67**	**96.18**
	CNN	86.21	38.78	73.48	82.92	52.02	84.38	7.56	80.62	72.40	65.38	98.25	82.82	87.94	92.30	91.63
	LSTM	83.66	28.76	74.96	**83.98**	55.44	90.44	8.12	81.88	75.96	62.66	99.17	84.52	91.31	96.09	93.75
	BiLSTM	89.92	39.72	75.06	80.52	56.02	87.72	13.14	79.64	81.02	62.96	99.24	84.52	91.28	95.89	93.33
	CNN-LSTM	**90.52**	42.81	76.32	82.25	59.18	88.76	13.56	**82.34**	78.86	68.73	99.23	87.46	91.39	95.13	95.46
	CNN-BiLSTM	88.04	45.96	76.26	82.44	60.84	89.92	11.72	82.18	**79.62**	67.68	99.26	86.43	91.41	96.40	95.44

**FIGURE 5:**
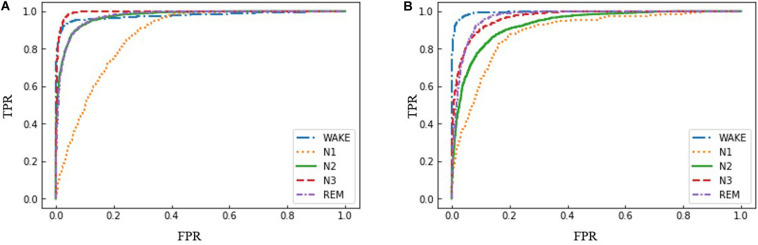
ROC curves for sleep stages using the proposed AT-BiLSTM models trained with **(A)** Fpz-Cz channel of PSEE dataset and **(B)** Fp1-A1 channel of DRM-SUB dataset.

Furthermore, ROC curves were used to compare the performances of the proposed AT-BiLSTM model for different sleep stages with the frontal channels in both datasets (Figure 5). As shown in Figure 5, AT-BiLSTM is sufficient to identify WAKE, N3 and REM, but insufficient to identify N1.

Table 7 illustrates the results of a comparison between the proposed AT-BiLSTM model and the state-of-the-art works using the same dataset of DRM-SUB (Hassan and Bhuiyan, 2016a,b; Hassan and Subasi, 2017; Ghimatgar et al., 2019; Shen et al., 2019). With the same dataset, same EEG channel and same dataset splitting strategy, the proposed AT-BiLSTM model achieves the highest accuracy.

**TABLE 7:** Comparison of sleep staging performance on the DRM-SUB dataset between the proposed method and previous works based on conventional feature extraction.

Authors	Year	Methodology	Dataset splitting strategy	Channel	Accuracy
Hassan and Bhuiyan, 2016a	2016	Tunable Q-factor wavelet transform, random forest (Hassan and Bhuiyan, 2016a)	Epoch-wise	Fp1-A1	72.28%
Hassan and Bhuiyan, 2016b	2016	Implementation of ensemble empirical mode decomposition in conjunction with random under sampling boosting (Hassan and Bhuiyan, 2016a)	Epoch-wise	Fp1-A1	74.59%
Hassan and Subasi, 2017	2017	Tunable Q-factor wavelet transform, bagging (Hassan and Subasi, 2017)	Epoch-wise	Fp1-A1	78.95%
Shen et al.	2019	Essence features extraction method (Shen et al., 2019)	Subject-wise	Cz-A1	80.90%
Ghimatgar et al.	2019	Features in time domain, frequency domain, cepstral domain, wavelet features, autoregressive coefficients and non-linear features with Hidden Markov Model (Ghimatgar et al., 2019)	Subject-wise	Fp1-A1	81.22%
Proposed method		Raw EEG signal and AT-BiLSTM	Subject-wise	Fp1-A1	81.72%

## Discussion

In this study, we proposed an AT-BiLSTM network for automatic sleep staging with single-channel EEG. The main findings were: (1) the frontal EEG derivations contribute to better performance of sleep staging than those located in the central, occipital or parietal lobe; (2) the proposed AT-BiLSTM network outperforms the other networks based on CNN or RNN; (3) The proposed deep learning network achieves higher accuracy than conventional feature extraction methods.

Two EEG datasets, i.e., PSEE and DRM-SUB, with different EEG derivations were used in our study. To clarify the influence of the EEG channel on automatic sleep staging, here we applied the proposed method to all the EEG channels in both datasets. The results obtained from both datasets are similar: the model adopting frontal derivation behaved better than those from other lobes. Such a finding indicated that the performance of sleep scoring was sensitive to the selection of EEG channel and the derivations from the frontal region are the optimal choices. Physiologically, the prefrontal cortex is deactivated and reactivated during the sleep cycle, indicating its involvement in the wake–sleep cycle (Maquet et al., 1996). With the development of wearable EEG devices, EEG signals can be easily obtained using dry electrodes on the forehead (Hassan and Bhuiyan, 2016a); the proposed method would be promising in supporting people monitoring sleep.

In recent years, many automated sleep staging methods based on deep neural networks used CNNs for feature extraction and RNNs to capture temporal information. These approaches have significantly improved the accuracy of sleep staging (Hassan and Bhuiyan, 2016a; Boostani et al., 2017; Sors et al., 2018). In general, for the sequence-to-label model based on RNN, only the output vector at the last time step is retained for classification, e.g., *via* a softmax layer (Phan et al., 2017). However, it is reasonable to combine the output vectors of different time steps by some weighting schemes. Intuitively, those parts of the input sequence which are essential to the classification task at hand should be associated with strong weights, and those with less importance should be weighted correspondingly less. Ideally, these weights should be automatically learned by the network. This can be accomplished with an attention layer (Luong et al., 2015). Besides, previous works demonstrated that the performance of classification or regression can be further improved by stacking multiple BiLSTM in neural networks (Liu et al., 2017; Wang et al., 2018; Liu et al., 2018). Aside from that, we found the overall performance of the RNN based model to be better than that of the CNN models in automatic sleep staging, which might indicate that the RNNs are promising in capturing the temporal nature of an EEG time series. From such a perspective, the highest performance achieved by the proposed AT-BiLSTM might further confirm the role of stacking layers and attention mechanism in feature extracting of time series.

In this study, all experiments were performed on a server configured with 64 CPUs [Intel(R) Xeon(R) CPU @ 2.10 GHz), 64 GB memory, a GPU (NVIDIA GeForce GTX 1,080 Ti] and a Windows Server 2016 system. A CNN network has the lowest computational cost as its training time for each batch was 0.16 s on average. LSTM and CNN-LSTM networks take similar times (8.46 and 8.60 s respectively) for each batch in training. The computational cost of BiLSTM based networks is twice that of LSTM based networks because they must calculate the input sequence in two directions and set up double parameters. Moreover, approximately 1.3 s more is required for each batch with the attention layer.

Our study demonstrated that a deep learning approach without manual feature extraction can also provide sufficient accuracy for sleep staging, which is even better than conventional methods based on manual feature extraction. Therefore, the proposed method is a promising choice for computer-aided detection of sleep stages and similar 1-D signal classification problems. In conclusion, our findings provide a possible solution for automatic sleep scoring without manual signal preprocessing and feature extraction. With the development of wearable EEG devices, such a solution would be valuable in the screening of sleep disorders at home for the general population.

## Data Availability Statement

The datasets presented in this study can be found in online repositories. The names of the repository/repositories and accession number(s) can be found below: Dreams Subjects: https://zenodo.org/record/2650142#.X6tbymgzZdg. Sleep-EDF Database Expanded: https://physionet.org/content/sleep-edfx/1.0.0/.

## Ethics Statement

The studies involving human participants were reviewed and approved by the institutional review board of two open-access databsets, i.e., the Sleep-EDF Expanded dataset available at Physionet and the DREAMS Subjects dataset. The patients/participants provided their written informed consent to participate in this study.

## Author Contributions

FH, XL, FX, and JL designed this study. MF and YW analyzed the data. MF, FH, and ZC wrote the article. All authors contributed to the article and approved the submitted version.

## Conflict of Interest

The authors declare that the research was conducted in the absence of any commercial or financial relationships that could be construed as a potential conflict of interest.
